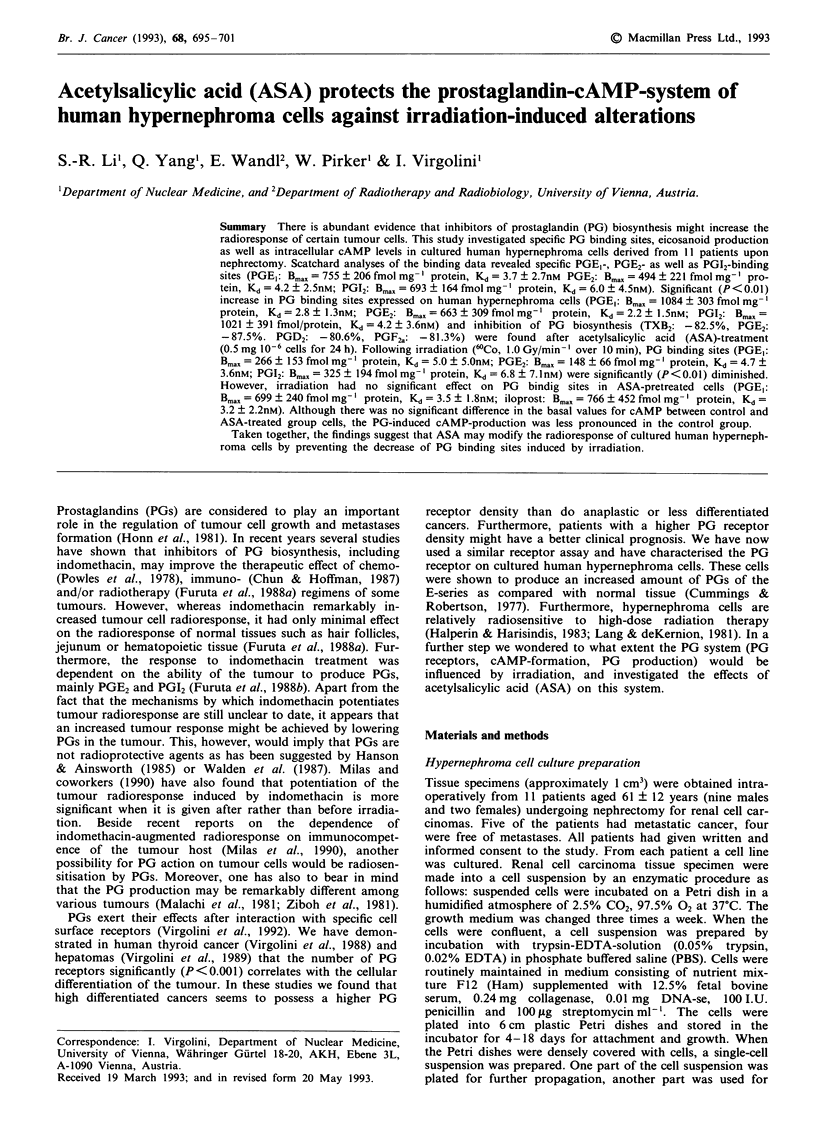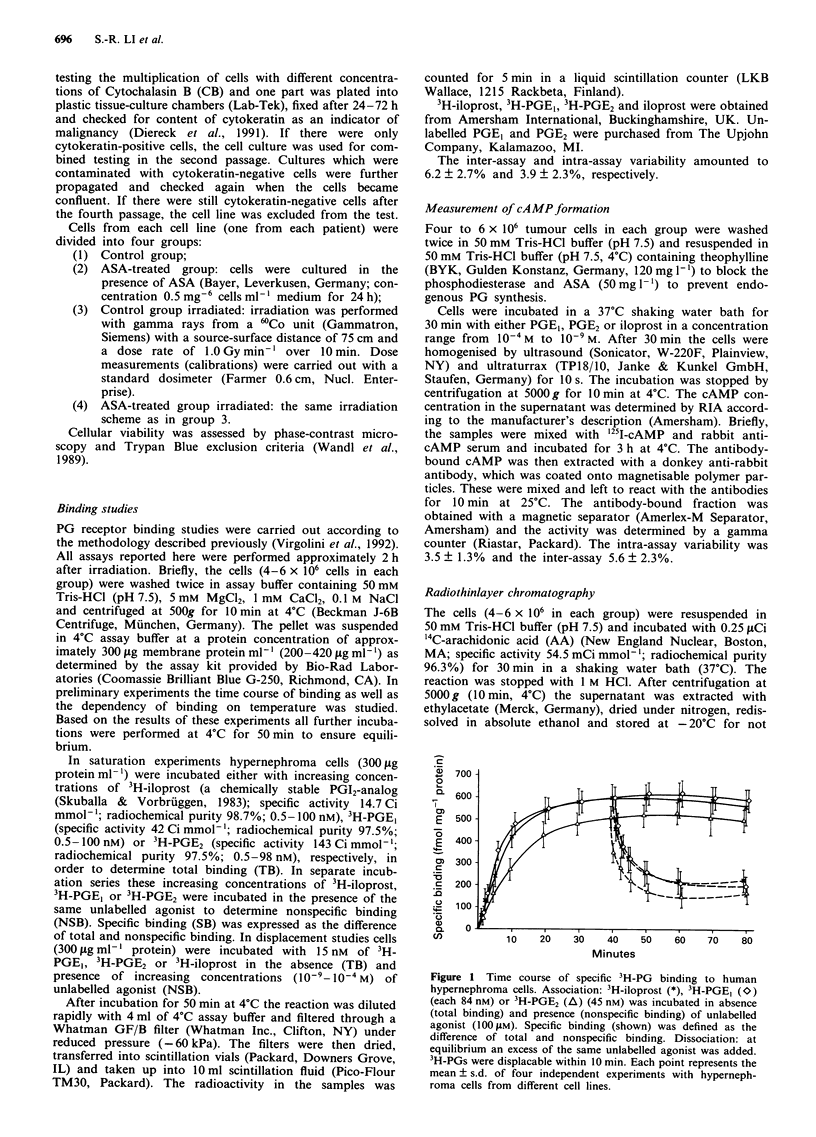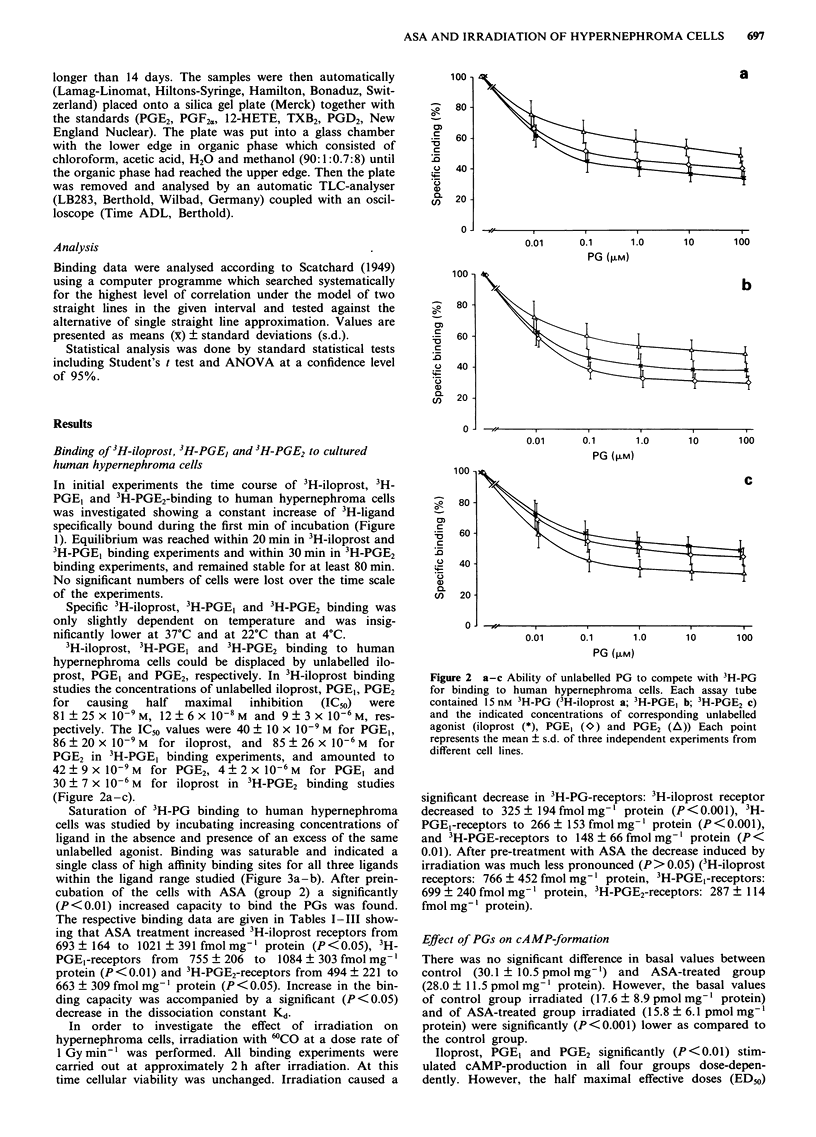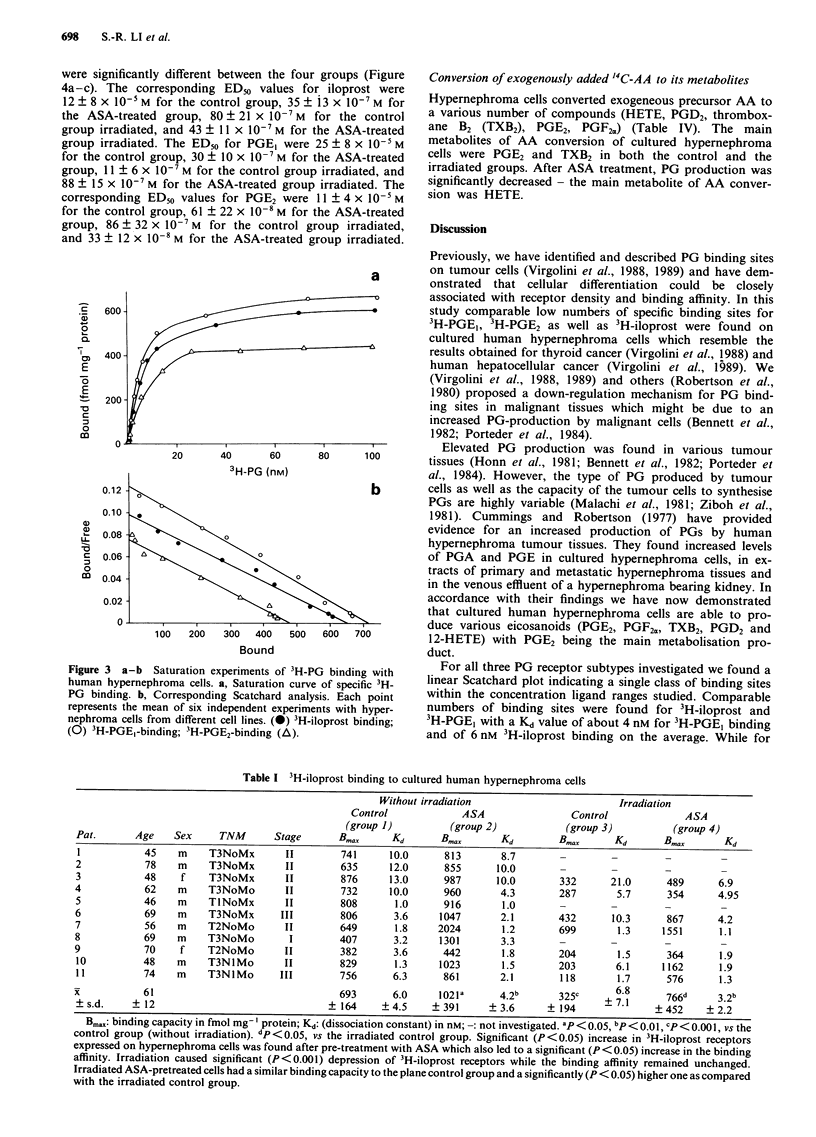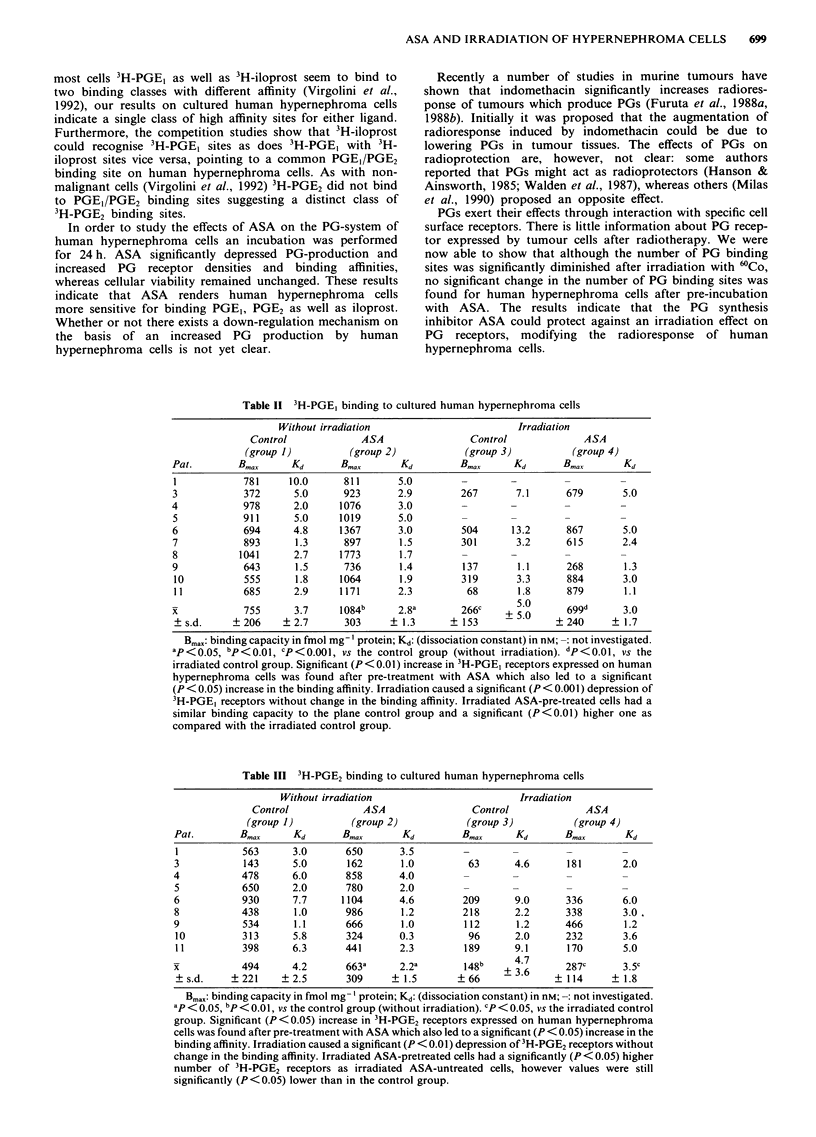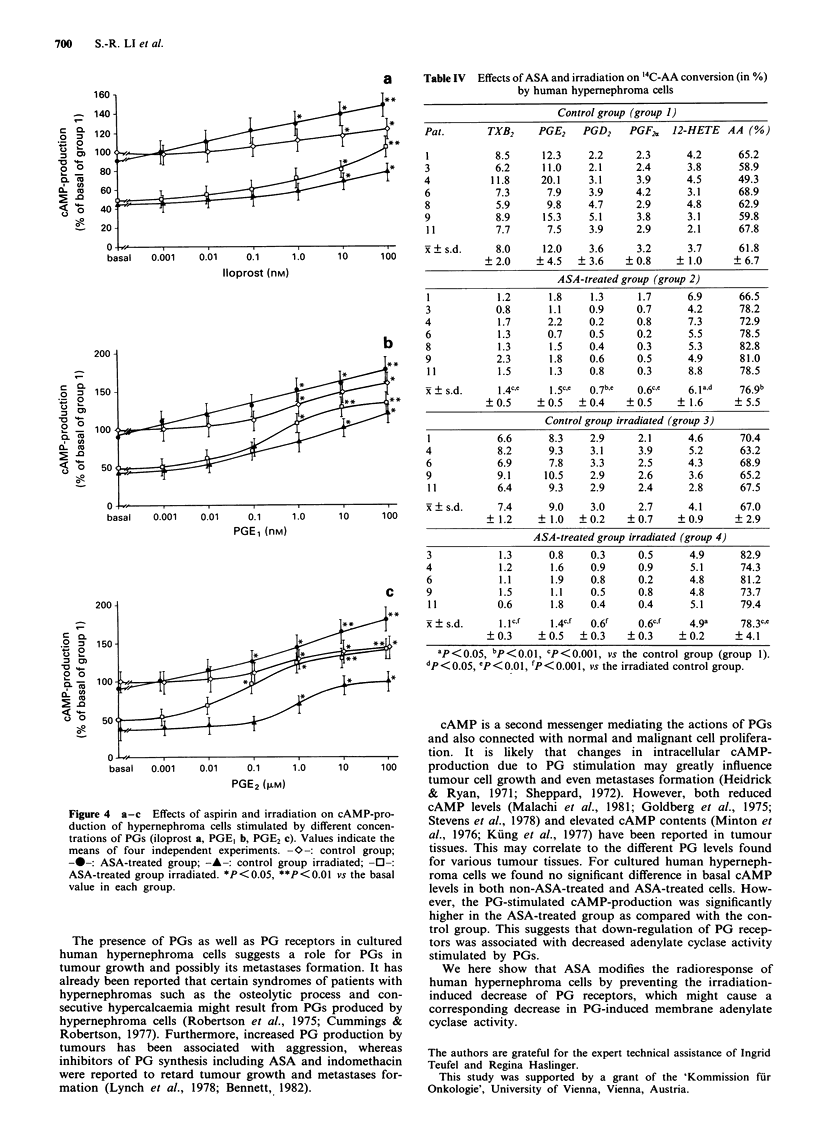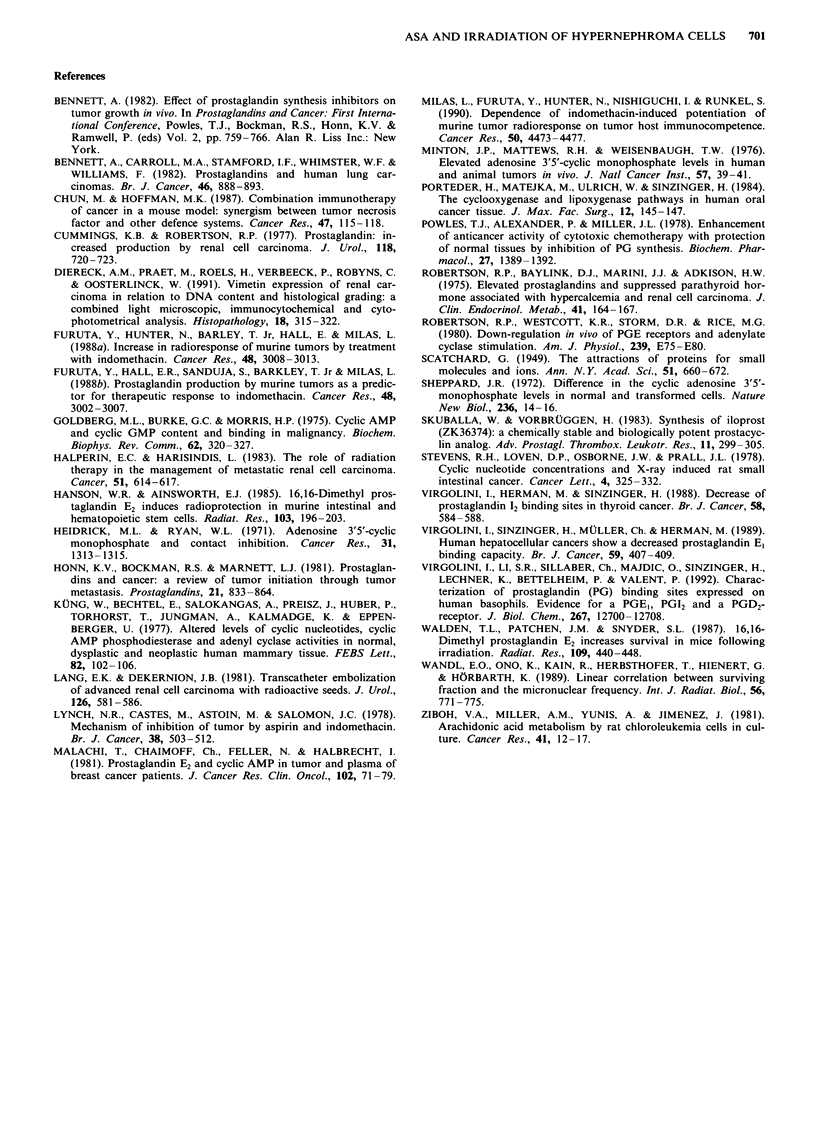# Acetylsalicylic acid (ASA) protects the prostaglandin-cAMP-system of human hypernephroma cells against irradiation-induced alterations.

**DOI:** 10.1038/bjc.1993.412

**Published:** 1993-10

**Authors:** S. R. Li, Q. Yang, E. Wandl, W. Pirker, I. Virgolini

**Affiliations:** Department of Nuclear Medicine, University of Vienna, Austria.

## Abstract

There is abundant evidence that inhibitors of prostaglandin (PG) biosynthesis might increase the radioresponse of certain tumour cells. This study investigated specific PG binding sites, eicosanoid production as well as intracellular cAMP levels in cultured human hypernephroma cells derived from 11 patients upon nephrectomy. Scatchard analyses of the binding data revealed specific PGE1-, PGE2- as well as PGI2-binding sites (PGE1: Bmax = 755 +/- 206 fmol mg-1 protein, Kd = 3.7 +/- 2.7 nM PGE2: Bmax = 494 +/- 221 fmol mg-1 protein, Kd = 4.2 +/- 2.5 nM; PGI2: Bmax = 693 +/- 164 fmol mg-1 protein, Kd = 6.0 +/- 4.5 nM). Significant (P < 0.01) increase in PG binding sites expressed on human hypernephroma cells (PGE1: Bmax = 1084 +/- 303 fmol mg-1 protein, Kd = 2.8 +/- 1.3 nM; PGE2: Bmax = 663 +/- 309 fmol mg-1 protein, Kd = 2.2 +/- 1.5 nM; PGI2: Bmax = 1021 +/- 391 fmol/protein, Kd = 4.2 +/- 3.6 nM) and inhibition of PG biosynthesis (TXB2: -82.5%, PGE2: -87.5%. PGD2: -80.6%, PGF2: -81.3%) were found after acetylsalicylic acid (ASA)-treatment (0.5 mg 10(-6) cells for 24 h). Following irradiation (60Co, 1.0 Gy/min-1 over 10(min), PG binding sites (PGE1: Bmax = 266 +/- 153 fmol mg-1 protein, Kd = 5.0 +/- 5.0 nM; PGE2: Bmax = 148 +/- 66 fmol mg-1 protein, Kd = 4.7 +/- 3.6 nM; PGI2: Bmax = 325 +/- 194 fmol mg-1 protein, Kd = 6.8 +/- 7.1 nM) were significantly (P < 0.01) diminished. However, irradiation had no significant effect on PG binding sites in ASA-pretreated cells (PGE1: Bmax = 699 +/- 240 fmol mg-1 protein, Kd = 3.5 +/- 1.8 nM; iloprost: Bmax = 766 +/- 452 fmol mg-1 protein, Kd = 3.2 +/- 2.2 nM). Although there was no significant difference in the basal values for cAMP between control and ASA-treated group cells, the PG-induced cAMP-production was less pronounced in the control group. Taken together, the findings suggest that ASA may modify the radioresponse of cultured human hypernephroma cells by preventing the decrease of PG binding sites induced by irradiation.


Br. J. Cancer (1993), 68, 695–701
© Macmillan Press Ltd., 1993

### Acetylsalicylic acid (ASA) protects the prostaglandin-cAMP-system of human hypernephroma cells against irradiation-induced alterations

S.-R. Li[1], Q. Yang[1], E. Wandl[2], W. Pirker[1] & I. Virgolini[1]

[1]*Department of Nuclear Medicine, and* [2]*Department of Radiotherapy and Radiobiology, University of Vienna, Austria.*

**Summary** There is abundant evidence that inhibitors of prostaglandin (PG) biosynthesis might increase the radioresponse of certain tumour cells. This study investigated specific PG binding sites, eicosanoid production as well as intracellular cAMP levels in cultured human hypernephroma cells derived from 11 patients upon nephrectomy. Scatchard analyses of the binding data revealed specific $PGE_1$-, $PGE_2$- as well as $PGI_2$-binding sites ($PGE_1$: $B_{max} = 755 \pm 206$ fmol mg$^{-1}$ protein, $K_d = 3.7 \pm 2.7$nM $PGE_2$: $B_{max} = 494 \pm 221$ fmol mg$^{-1}$ protein, $K_d = 4.2 \pm 2.5$nM; $PGI_2$: $B_{max} = 693 \pm 164$ fmol mg$^{-1}$ protein, $K_d = 6.0 \pm 4.5$nM). Significant ($P < 0.01$) increase in PG binding sites expressed on human hypernephroma cells ($PGE_1$: $B_{max} = 1084 \pm 303$ fmol mg$^{-1}$ protein, $K_d = 2.8 \pm 1.3$nM; $PGE_2$: $B_{max} = 663 \pm 309$ fmol mg$^{-1}$ protein, $K_d = 2.2 \pm 1.5$nM; $PGI_2$: $B_{max} = 1021 \pm 391$ fmol/protein, $K_d = 4.2 \pm 3.6$nM) and inhibition of PG biosynthesis ($TXB_2$: −82.5%, $PGE_2$: −87.5%. $PGD_2$: −80.6%, $PGF_{2a}$: −81.3%) were found after acetylsalicylic acid (ASA)-treatment (0.5 mg $10^{-6}$ cells for 24 h). Following irradiation ($^{60}$Co, 1.0 Gy/min$^{-1}$ over 10 min), PG binding sites ($PGE_1$: $B_{max} = 266 \pm 153$ fmol mg$^{-1}$ protein, $K_d = 5.0 \pm 5.0$nM; $PGE_2$: $B_{max} = 148 \pm 66$ fmol mg$^{-1}$ protein, $K_d = 4.7 \pm 3.6$nM; $PGI_2$: $B_{max} = 325 \pm 194$ fmol mg$^{-1}$ protein, $K_d = 6.8 \pm 7.1$nM) were significantly ($P < 0.01$) diminished. However, irradiation had no significant effect on PG bindig sites in ASA-pretreated cells ($PGE_1$: $B_{max} = 699 \pm 240$ fmol mg$^{-1}$ protein, $K_d = 3.5 \pm 1.8$nM; iloprost: $B_{max} = 766 \pm 452$ fmol mg$^{-1}$ protein, $K_d = 3.2 \pm 2.2$nM). Although there was no significant difference in the basal values for cAMP between control and ASA-treated group cells, the PG-induced cAMP-production was less pronounced in the control group.

Taken together, the findings suggest that ASA may modify the radioresponse of cultured human hypernephroma cells by preventing the decrease of PG binding sites induced by irradiation.

Prostaglandins (PGs) are considered to play an important role in the regulation of tumour cell growth and metastases formation (Honn *et al.*, 1981). In recent years several studies have shown that inhibitors of PG biosynthesis, including indomethacin, may improve the therapeutic effect of chemo- (Powles *et al.*, 1978), immuno- (Chun & Hoffman, 1987) and/or radiotherapy (Furuta *et al.*, 1988*a*) regimens of some tumours. However, whereas indomethacin remarkably increased tumour cell radioresponse, it had only minimal effect on the radioresponse of normal tissues such as hair follicles, jejunum or hematopoietic tissue (Furuta *et al.*, 1988*a*). Furthermore, the response to indomethacin treatment was dependent on the ability of the tumour to produce PGs, mainly $PGE_2$ and $PGI_2$ (Furuta *et al.*, 1988*b*). Apart from the fact that the mechanisms by which indomethacin potentiates tumour radioresponse are still unclear to date, it appears that an increased tumour response might be achieved by lowering PGs in the tumour. This, however, would imply that PGs are not radioprotective agents as has been suggested by Hanson & Ainsworth (1985) or Walden *et al.* (1987). Milas and coworkers (1990) have also found that potentiation of the tumour radioresponse induced by indomethacin is more significant when it is given after rather than before irradiation. Beside recent reports on the dependence of indomethacin-augmented radioresponse on immunocompetence of the tumour host (Milas *et al.*, 1990), another possibility for PG action on tumour cells would be radiosensitisation by PGs. Moreover, one has also to bear in mind that the PG production may be remarkably different among various tumours (Malachi *et al.*, 1981; Ziboh *et al.*, 1981).

PGs exert their effects after interaction with specific cell surface receptors (Virgolini *et al.*, 1992). We have demonstrated in human thyroid cancer (Virgolini *et al.*, 1988) and hepatomas (Virgolini *et al.*, 1989) that the number of PG receptors significantly ($P < 0.001$) correlates with the cellular differentiation of the tumour. In these studies we found that high differentiated cancers seems to possess a higher PG receptor density than do anaplastic or less differentiated cancers. Furthermore, patients with a higher PG receptor density might have a better clinical prognosis. We have now used a similar receptor assay and have characterised the PG receptor on cultured human hypernephroma cells. These cells were shown to produce an increased amount of PGs of the E-series as compared with normal tissue (Cummings & Robertson, 1977). Furthermore, hypernephroma cells are relatively radiosensitive to high-dose radiation therapy (Halperin & Harisindis, 1983; Lang & deKernion, 1981). In a further step we wondered to what extent the PG system (PG receptors, cAMP-formation, PG production) would be influenced by irradiation, and investigated the effects of acetylsalicylic acid (ASA) on this system.

### Materials and methods

### *Hypernephroma cell culture preparation*

Tissue specimens (approximately 1 cm$^3$) were obtained intraoperatively from 11 patients aged $61 \pm 12$ years (nine males and two females) undergoing nephrectomy for renal cell carcinomas. Five of the patients had metastatic cancer, four were free of metastases. All patients had given written and informed consent to the study. From each patient a cell line was cultured. Renal cell carcinoma tissue specimen were made into a cell suspension by an enzymatic procedure as follows: suspended cells were incubated on a Petri dish in a humidified atmosphere of 2.5% $CO_2$, 97.5% $O_2$ at 37°C. The growth medium was changed three times a week. When the cells were confluent, a cell suspension was prepared by incubation with trypsin-EDTA-solution (0.05% trypsin, 0.02% EDTA) in phosphate buffered saline (PBS). Cells were routinely maintained in medium consisting of nutrient mixture F12 (Ham) supplemented with 12.5% fetal bovine serum, 0.24 mg collagenase, 0.01 mg DNA-se, 100 I.U. penicillin and 100 μg streptomycin ml$^{-1}$. The cells were plated into 6 cm plastic Petri dishes and stored in the incubator for 4–18 days for attachment and growth. When the Petri dishes were densely covered with cells, a single-cell suspension was prepared. One part of the cell suspension was plated for further propagation, another part was used for

Correspondence: I. Virgolini, Department of Nuclear Medicine, University of Vienna, Währinger Gürtel 18-20, AKH, Ebene 3L, A-1090 Vienna, Austria.
Received 19 March 1993; and in revised form 20 May 1993.

testing the multiplication of cells with different concentrations of Cytochalasin B (CB) and one part was plated into plastic tissue-culture chambers (Lab-Tek), fixed after 24–72 h and checked for content of cytokeratin as an indicator of malignancy (Diereck *et al.*, 1991). If there were only cytokeratin-positive cells, the cell culture was used for combined testing in the second passage. Cultures which were contaminated with cytokeratin-negative cells were further propagated and checked again when the cells became confluent. If there were still cytokeratin-negative cells after the fourth passage, the cell line was excluded from the test.

Cells from each cell line (one from each patient) were divided into four groups:

(1) Control group;
(2) ASA-treated group: cells were cultured in the presence of ASA (Bayer, Leverkusen, Germany; concentration 0.5 $mg^{-6}$ cells $ml^{-1}$ medium for 24 h);
(3) Control group irradiated: irradiation was performed with gamma rays from a $^{60}Co$ unit (Gammatron, Siemens) with a source-surface distance of 75 cm and a dose rate of 1.0 Gy $min^{-1}$ over 10 min. Dose measurements (calibrations) were carried out with a standard dosimeter (Farmer 0.6 cm, Nucl. Enterprise).
(4) ASA-treated group irradiated: the same irradiation scheme as in group 3.

Cellular viability was assessed by phase-contrast microscopy and Trypan Blue exclusion criteria (Wandl *et al.*, 1989).

*Binding studies*

PG receptor binding studies were carried out according to the methodology described previously (Virgolini *et al.*, 1992). All assays reported here were performed approximately 2 h after irradiation. Briefly, the cells ($4-6 \times 10^6$ cells in each group) were washed twice in assay buffer containing 50 mM Tris-HCl (pH 7.5), 5 mM $MgCl_2$, 1 mM $CaCl_2$, 0.1 M NaCl and centrifuged at 500*g* for 10 min at 4°C (Beckman J-6B Centrifuge, München, Germany). The pellet was suspended in 4°C assay buffer at a protein concentration of approximately 300 μg membrane protein $ml^{-1}$ (200–420 μg $ml^{-1}$) as determined by the assay kit provided by Bio-Rad Laboratories (Coomassie Brilliant Blue G-250, Richmond, CA). In preliminary experiments the time course of binding as well as the dependency of binding on temperature was studied. Based on the results of these experiments all further incubations were performed at 4°C for 50 min to ensure equilibrium.

In saturation experiments hypernephroma cells (300 μg protein $ml^{-1}$) were incubated either with increasing concentrations of $^3H$-iloprost (a chemically stable $PGI_2$-analog (Skuballa & Vorbrüggen, 1983); specific activity 14.7 Ci $mmol^{-1}$; radiochemical purity 98.7%; 0.5–100 nM), $^3H$-$PGE_1$ (specific activity 42 Ci $mmol^{-1}$; radiochemical purity 97.5%; 0.5–100 nM) or $^3H$-$PGE_2$ (specific activity 143 Ci $mmol^{-1}$; radiochemical purity 97.5%; 0.5–98 nM), respectively, in order to determine total binding (TB). In separate incubation series these increasing concentrations of $^3H$-iloprost, $^3H$-$PGE_1$ or $^3H$-$PGE_2$ were incubated in the presence of the same unlabelled agonist to determine nonspecific binding (NSB). Specific binding (SB) was expressed as the difference of total and nonspecific binding. In displacement studies cells (300 μg $ml^{-1}$ protein) were incubated with 15 nM of $^3H$-$PGE_1$, $^3H$-$PGE_2$ or $^3H$-iloprost in the absence (TB) and presence of increasing concentrations ($10^{-9}-10^{-4}$ M) of unlabelled agonist (NSB).

After incubation for 50 min at 4°C the reaction was diluted rapidly with 4 ml of 4°C assay buffer and filtered through a Whatman GF/B filter (Whatman Inc., Clifton, NY) under reduced pressure (−60 kPa). The filters were then dried, transferred into scintillation vials (Packard, Downers Grove, IL) and taken up into 10 ml scintillation fluid (Pico-Flour TM30, Packard). The radioactivity in the samples was counted for 5 min in a liquid scintillation counter (LKB Wallace, 1215 Rackbeta, Finland).

$^3H$-iloprost, $^3H$-$PGE_1$, $^3H$-$PGE_2$ and iloprost were obtained from Amersham International, Buckinghamshire, UK. Unlabelled $PGE_1$ and $PGE_2$ were purchased from The Upjohn Company, Kalamazoo, MI.

The inter-assay and intra-assay variability amounted to 6.2 ± 2.7% and 3.9 ± 2.3%, respectively.

*Measurement of cAMP formation*

Four to $6 \times 10^6$ tumour cells in each group were washed twice in 50 mM Tris-HCl buffer (pH 7.5) and resuspended in 50 mM Tris-HCl buffer (pH 7.5, 4°C) containing theophylline (BYK, Gulden Konstanz, Germany, 120 mg $l^{-1}$) to block the phosphodiesterase and ASA (50 mg $l^{-1}$) to prevent endogenous PG synthesis.

Cells were incubated in a 37°C shaking water bath for 30 min with either $PGE_1$, $PGE_2$ or iloprost in a concentration range from $10^{-4}$ M to $10^{-9}$ M. After 30 min the cells were homogenised by ultrasound (Sonicator, W-220F, Plainview, NY) and ultraturrax (TP18/10, Janke & Kunkel GmbH, Staufen, Germany) for 10 s. The incubation was stopped by centrifugation at 5000 *g* for 10 min at 4°C. The cAMP concentration in the supernatant was determined by RIA according to the manufacturer's description (Amersham). Briefly, the samples were mixed with $^{125}I$-cAMP and rabbit anti-cAMP serum and incubated for 3 h at 4°C. The antibody-bound cAMP was then extracted with a donkey anti-rabbit antibody, which was coated onto magnetisable polymer particles. These were mixed and left to react with the antibodies for 10 min at 25°C. The antibody-bound fraction was obtained with a magnetic separator (Amerlex-M Separator, Amersham) and the activity was determined by a gamma counter (Riastar, Packard). The intra-assay variability was 3.5 ± 1.3% and the inter-assay 5.6 ± 2.3%.

*Radiothinlayer chromatography*

The cells ($4-6 \times 10^6$ in each group) were resuspended in 50 mM Tris-HCl buffer (pH 7.5) and incubated with 0.25 μCi $^{14}C$-arachidonic acid (AA) (New England Nuclear, Boston, MA; specific activity 54.5 mCi $mmol^{-1}$; radiochemical purity 96.3%) for 30 min in a shaking water bath (37°C). The reaction was stopped with 1 M HCl. After centrifugation at 5000 *g* (10 min, 4°C) the supernatant was extracted with ethylacetate (Merck, Germany), dried under nitrogen, redissolved in absolute ethanol and stored at −20°C for not

**Figure 1** Time course of specific $^3H$-PG binding to human hypernephroma cells. Association: $^3H$-iloprost (*), $^3H$-$PGE_1$ (◇) (each 84 nM) or $^3H$-$PGE_2$ (Δ) (45 nM) was incubated in absence (total binding) and presence (nonspecific binding) of unlabelled agonist (100 μM). Specific binding (shown) was defined as the difference of total and nonspecific binding. Dissociation: at equilibrium an excess of the same unlabelled agonist was added. $^3H$-PGs were displacable within 10 min. Each point represents the mean ± s.d. of four independent experiments with hypernephroma cells from different cell lines.

longer than 14 days. The samples were then automatically (Lamag-Linomat, Hiltons-Syringe, Hamilton, Bonaduz, Switzerland) placed onto a silica gel plate (Merck) together with the standards ($PGE_2$, $PGF_{2\alpha}$, 12-HETE, $TXB_2$, $PGD_2$, New England Nuclear). The plate was put into a glass chamber with the lower edge in organic phase which consisted of chloroform, acetic acid, $H_2O$ and methanol (90:1:0.7:8) until the organic phase had reached the upper edge. Then the plate was removed and analysed by an automatic TLC-analyser (LB283, Berthold, Wilbad, Germany) coupled with an oscilloscope (Time ADL, Berthold).

*Analysis*

Binding data were analysed according to Scatchard (1949) using a computer programme which searched systematically for the highest level of correlation under the model of two straight lines in the given interval and tested against the alternative of single straight line approximation. Values are presented as means ($\bar{x}$) ± standard deviations (s.d.).

Statistical analysis was done by standard statistical tests including Student's *t* test and ANOVA at a confidence level of 95%.

### Results

*Binding of $^3H$-iloprost, $^3H$-$PGE_1$ and $^3H$-$PGE_2$ to cultured human hypernephroma cells*

In initial experiments the time course of $^3H$-iloprost, $^3H$-$PGE_1$ and $^3H$-$PGE_2$-binding to human hypernephroma cells was investigated showing a constant increase of $^3H$-ligand specifically bound during the first min of incubation (Figure 1). Equilibrium was reached within 20 min in $^3H$-iloprost and $^3H$-$PGE_1$ binding experiments and within 30 min in $^3H$-$PGE_2$ binding experiments, and remained stable for at least 80 min. No significant numbers of cells were lost over the time scale of the experiments.

Specific $^3H$-iloprost, $^3H$-$PGE_1$ and $^3H$-$PGE_2$ binding was only slightly dependent on temperature and was insignificantly lower at 37°C and at 22°C than at 4°C.

$^3H$-iloprost, $^3H$-$PGE_1$ and $^3H$-$PGE_2$ binding to human hypernephroma cells could be displaced by unlabelled iloprost, $PGE_1$ and $PGE_2$, respectively. In $^3H$-iloprost binding studies the concentrations of unlabelled iloprost, $PGE_1$, $PGE_2$ for causing half maximal inhibition ($IC_{50}$) were $81 \pm 25 \times 10^{-9}$ M, $12 \pm 6 \times 10^{-8}$ M and $9 \pm 3 \times 10^{-6}$ M, respectively. The $IC_{50}$ values were $40 \pm 10 \times 10^{-9}$ M for $PGE_1$, $86 \pm 20 \times 10^{-9}$ M for iloprost, and $85 \pm 26 \times 10^{-6}$ M for $PGE_2$ in $^3H$-$PGE_1$ binding experiments, and amounted to $42 \pm 9 \times 10^{-9}$ M for $PGE_2$, $4 \pm 2 \times 10^{-6}$ M for $PGE_1$ and $30 \pm 7 \times 10^{-6}$ M for iloprost in $^3H$-$PGE_2$ binding studies (Figure 2a–c).

Saturation of $^3H$-PG binding to human hypernephroma cells was studied by incubating increasing concentrations of ligand in the absence and presence of an excess of the same unlabelled agonist. Binding was saturable and indicated a single class of high affinity binding sites for all three ligands within the ligand range studied (Figure 3a–b). After preincubation of the cells with ASA (group 2) a significantly ($P < 0.01$) increased capacity to bind the PGs was found. The respective binding data are given in Tables I–III showing that ASA treatment increased $^3H$-iloprost receptors from 693 ± 164 to 1021 ± 391 fmol $mg^{-1}$ protein ($P < 0.05$), $^3H$-$PGE_1$-receptors from 755 ± 206 to 1084 ± 303 fmol $mg^{-1}$ protein ($P < 0.01$) and $^3H$-$PGE_2$-receptors from 494 ± 221 to 663 ± 309 fmol $mg^{-1}$ protein ($P < 0.05$). Increase in the binding capacity was accompanied by a significant ($P < 0.05$) decrease in the dissociation constant $K_d$.

In order to investigate the effect of irradiation on hypernephroma cells, irradiation with $^{60}CO$ at a dose rate of 1 Gy $min^{-1}$ was performed. All binding experiments were carried out at approximately 2 h after irradiation. At this time cellular viability was unchanged. Irradiation caused a significant decrease in $^3H$-PG-receptors: $^3H$-iloprost receptor decreased to 325 ± 194 fmol $mg^{-1}$ protein ($P < 0.001$), $^3H$-$PGE_1$-receptors to 266 ± 153 fmol $mg^{-1}$ protein ($P < 0.001$), and $^3H$-PGE-receptors to 148 ± 66 fmol $mg^{-1}$ protein ($P < 0.01$). After pre-treatment with ASA the decrease induced by irradiation was much less pronounced ($P > 0.05$) ($^3H$-iloprost receptors: 766 ± 452 fmol $mg^{-1}$ protein, $^3H$-$PGE_1$-receptors: 699 ± 240 fmol $mg^{-1}$ protein, $^3H$-$PGE_2$-receptors: 287 ± 114 fmol $mg^{-1}$ protein).

**Figure 2** a–c Ability of unlabelled PG to compete with $^3H$-PG for binding to human hypernephroma cells. Each assay tube contained 15 nM $^3H$-PG ($^3H$-iloprost **a**; $^3H$-$PGE_1$ **b**; $^3H$-$PGE_2$ **c**) and the indicated concentrations of corresponding unlabelled agonist (iloprost (*), $PGE_1$ (◇) and $PGE_2$ (△)) Each point represents the mean ± s.d. of three independent experiments from different cell lines.

*Effect of PGs on cAMP-formation*

There was no significant difference in basal values between control (30.1 ± 10.5 pmol $mg^{-1}$) and ASA-treated group (28.0 ± 11.5 pmol $mg^{-1}$ protein). However, the basal values of control group irradiated (17.6 ± 8.9 pmol $mg^{-1}$ protein) and of ASA-treated group irradiated (15.8 ± 6.1 pmol $mg^{-1}$ protein) were significantly ($P < 0.001$) lower as compared to the control group.

Iloprost, $PGE_1$ and $PGE_2$ significantly ($P < 0.01$) stimulated cAMP-production in all four groups dose-dependently. However, the half maximal effective doses ($ED_{50}$)

were significantly different between the four groups (Figure 4a–c). The corresponding $ED_{50}$ values for iloprost were $12 \pm 8 \times 10^{-5}$ M for the control group, $35 \pm 13 \times 10^{-7}$ M for the ASA-treated group, $80 \pm 21 \times 10^{-7}$ M for the control group irradiated, and $43 \pm 11 \times 10^{-7}$ M for the ASA-treated group irradiated. The $ED_{50}$ for $PGE_1$ were $25 \pm 8 \times 10^{-5}$ M for the control group, $30 \pm 10 \times 10^{-7}$ M for the ASA-treated group, $11 \pm 6 \times 10^{-7}$ M for the control group irradiated, and $88 \pm 15 \times 10^{-7}$ M for the ASA-treated group irradiated. The corresponding $ED_{50}$ values for $PGE_2$ were $11 \pm 4 \times 10^{-5}$ M for the control group, $61 \pm 22 \times 10^{-8}$ M for the ASA-treated group, $86 \pm 32 \times 10^{-7}$ M for the control group irradiated, and $33 \pm 12 \times 10^{-8}$ M for the ASA-treated group irradiated.

**Figure 3 a–b** Saturation experiments of $^3$H-PG binding with human hypernephroma cells. **a**, Saturation curve of specific $^3$H-PG binding. **b**, Corresponding Scatchard analysis. Each point represents the mean of six independent experiments with hypernephroma cells from different cell lines. (●) $^3$H-iloprost binding; (○) $^3$H-$PGE_1$-binding; $^3$H-$PGE_2$-binding (△).

*Conversion of exogenously added $^{14}$C-AA to its metabolites*

Hypernephroma cells converted exogeneous precursor AA to a various number of compounds (HETE, $PGD_2$, thromboxane $B_2$ ($TXB_2$), $PGE_2$, $PGF_{2\alpha}$) (Table IV). The main metabolites of AA conversion of cultured hypernephroma cells were $PGE_2$ and $TXB_2$ in both the control and the irradiated groups. After ASA treatment, PG production was significantly decreased – the main metabolite of AA conversion was HETE.

### Discussion

Previously, we have identified and described PG binding sites on tumour cells (Virgolini *et al.*, 1988, 1989) and have demonstrated that cellular differentiation could be closely associated with receptor density and binding affinity. In this study comparable low numbers of specific binding sites for $^3$H-$PGE_1$, $^3$H-$PGE_2$ as well as $^3$H-iloprost were found on cultured human hypernephroma cells which resemble the results obtained for thyroid cancer (Virgolini *et al.*, 1988) and human hepatocellular cancer (Virgolini *et al.*, 1989). We (Virgolini *et al.*, 1988, 1989) and others (Robertson *et al.*, 1980) proposed a down-regulation mechanism for PG binding sites in malignant tissues which might be due to an increased PG-production by malignant cells (Bennett *et al.*, 1982; Porteder *et al.*, 1984).

Elevated PG production was found in various tumour tissues (Honn *et al.*, 1981; Bennett *et al.*, 1982; Porteder *et al.*, 1984). However, the type of PG produced by tumour cells as well as the capacity of the tumour cells to synthesise PGs are highly variable (Malachi *et al.*, 1981; Ziboh *et al.*, 1981). Cummings and Robertson (1977) have provided evidence for an increased production of PGs by human hypernephroma tumour tissues. They found increased levels of PGA and PGE in cultured hypernephroma cells, in extracts of primary and metastatic hypernephroma tissues and in the venous effluent of a hypernephroma bearing kidney. In accordance with their findings we have now demonstrated that cultured human hypernephroma cells are able to produce various eicosanoids ($PGE_2$, $PGF_{2\alpha}$, $TXB_2$, $PGD_2$ and 12-HETE) with $PGE_2$ being the main metabolisation product.

For all three PG receptor subtypes investigated we found a linear Scatchard plot indicating a single class of binding sites within the concentration ligand ranges studied. Comparable numbers of binding sites were found for $^3$H-iloprost and $^3$H-$PGE_1$ with a $K_d$ value of about 4 nM for $^3$H-$PGE_1$ binding and of 6 nM $^3$H-iloprost binding on the average. While for

**Table I** $^3$H-iloprost binding to cultured human hypernephroma cells

| | | | | | Without irradiation | | | | Irradiation | | | |
|---|---|---|---|---|---|---|---|---|---|---|---|---|
| | | | | | Control (group 1) | | ASA (group 2) | | Control (group 3) | | ASA (group 4) | |
| Pat. | Age | Sex | TNM | Stage | $B_{max}$ | $K_d$ | $B_{max}$ | $K_d$ | $B_{max}$ | $K_d$ | $B_{max}$ | $K_d$ |
| 1 | 45 | m | T3NoMx | II | 741 | 10.0 | 813 | 8.7 | – | – | – | – |
| 2 | 78 | m | T3NoMx | II | 635 | 12.0 | 855 | 10.0 | – | – | – | – |
| 3 | 48 | f | T3NoMx | II | 876 | 13.0 | 987 | 10.0 | 332 | 21.0 | 489 | 6.9 |
| 4 | 62 | m | T3NoMo | II | 732 | 10.0 | 960 | 4.3 | 287 | 5.7 | 354 | 4.95 |
| 5 | 46 | m | T1NoMx | II | 808 | 1.0 | 916 | 1.0 | – | – | – | – |
| 6 | 69 | m | T3NoMx | III | 806 | 3.6 | 1047 | 2.1 | 432 | 10.3 | 867 | 4.2 |
| 7 | 56 | m | T2NoMo | II | 649 | 1.8 | 2024 | 1.2 | 699 | 1.3 | 1551 | 1.1 |
| 8 | 69 | m | T3NoMo | I | 407 | 3.2 | 1301 | 3.3 | – | – | – | – |
| 9 | 70 | f | T2NoMo | II | 382 | 3.6 | 442 | 1.8 | 204 | 1.5 | 364 | 1.9 |
| 10 | 48 | m | T3N1Mo | II | 829 | 1.3 | 1023 | 1.5 | 203 | 6.1 | 1162 | 1.9 |
| 11 | 74 | m | T3N1Mo | III | 756 | 6.3 | 861 | 2.1 | 118 | 1.7 | 576 | 1.3 |
| x̄ | 61 | | | | 693 | 6.0 | 1021[a] | 4.2[b] | 325[c] | 6.8 | 766[d] | 3.2[b] |
| ± s.d. | ± 12 | | | | ± 164 | ± 4.5 | ± 391 | ± 3.6 | ± 194 | ± 7.1 | ± 452 | ± 2.2 |

$B_{max}$: binding capacity in fmol mg$^{-1}$ protein; $K_d$: (dissociation constant) in nM; –: not investigated. [a]$P < 0.05$, [b]$P < 0.01$, [c]$P < 0.001$, *vs* the control group (without irradiation). [d]$P < 0.05$, *vs* the irradiated control group. Significant ($P < 0.05$) increase in $^3$H-iloprost receptors expressed on hypernephroma cells was found after pre-treatment with ASA which also led to a significant ($P < 0.05$) increase in the binding affinity. Irradiation caused significant ($P < 0.001$) depression of $^3$H-iloprost receptors while the binding affinity remained unchanged. Irradiated ASA-pretreated cells had a similar binding capacity to the plane control group and a significantly ($P < 0.05$) higher one as compared with the irradiated control group.

most cells $^3$H-PGE$_1$ as well as $^3$H-iloprost seem to bind to two binding classes with different affinity (Virgolini *et al.*, 1992), our results on cultured human hypernephroma cells indicate a single class of high affinity sites for either ligand. Furthermore, the competition studies show that $^3$H-iloprost could recognise $^3$H-PGE$_1$ sites as does $^3$H-PGE$_1$ with $^3$H-iloprost sites vice versa, pointing to a common PGE$_1$/PGE$_2$ binding site on human hypernephroma cells. As with non-malignant cells (Virgolini *et al.*, 1992) $^3$H-PGE$_2$ did not bind to PGE$_1$/PGE$_2$ binding sites suggesting a distinct class of $^3$H-PGE$_2$ binding sites.

In order to study the effects of ASA on the PG-system of human hypernephroma cells an incubation was performed for 24 h. ASA significantly depressed PG-production and increased PG receptor densities and binding affinities, whereas cellular viability remained unchanged. These results indicate that ASA renders human hypernephroma cells more sensitive for binding PGE$_1$, PGE$_2$ as well as iloprost. Whether or not there exists a down-regulation mechanism on the basis of an increased PG production by human hypernephroma cells is not yet clear.

Recently a number of studies in murine tumours have shown that indomethacin significantly increases radioresponse of tumours which produce PGs (Furuta *et al.*, 1988*a*, 1988*b*). Initially it was proposed that the augmentation of radioresponse induced by indomethacin could be due to lowering PGs in tumour tissues. The effects of PGs on radioprotection are, however, not clear: some authors reported that PGs might act as radioprotectors (Hanson & Ainsworth, 1985; Walden *et al.*, 1987), whereas others (Milas *et al.*, 1990) proposed an opposite effect.

PGs exert their effects through interaction with specific cell surface receptors. There is little information about PG receptor expressed by tumour cells after radiotherapy. We were now able to show that although the number of PG binding sites was significantly diminished after irradiation with $^{60}$Co, no significant change in the number of PG binding sites was found for human hypernephroma cells after pre-incubation with ASA. The results indicate that the PG synthesis inhibitor ASA could protect against an irradiation effect on PG receptors, modifying the radioresponse of human hypernephroma cells.

**Table II** $^3$H-PGE$_1$ binding to cultured human hypernephroma cells

| | *Without irradiation* | | | | *Irradiation* | | | |
|---|---|---|---|---|---|---|---|---|
| | *Control (group 1)* | | *ASA (group 2)* | | *Control (group 3)* | | *ASA (group 4)* | |
| *Pat.* | $B_{max}$ | $K_d$ | $B_{max}$ | $K_d$ | $B_{max}$ | $K_d$ | $B_{max}$ | $K_d$ |
| 1 | 781 | 10.0 | 811 | 5.0 | – | – | – | – |
| 3 | 372 | 5.0 | 923 | 2.9 | 267 | 7.1 | 679 | 5.0 |
| 4 | 978 | 2.0 | 1076 | 3.0 | – | – | – | – |
| 5 | 911 | 5.0 | 1019 | 5.0 | – | – | – | – |
| 6 | 694 | 4.8 | 1367 | 3.0 | 504 | 13.2 | 867 | 5.0 |
| 7 | 893 | 1.3 | 897 | 1.5 | 301 | 3.2 | 615 | 2.4 |
| 8 | 1041 | 2.7 | 1773 | 1.7 | – | – | – | – |
| 9 | 643 | 1.5 | 736 | 1.4 | 137 | 1.1 | 268 | 1.3 |
| 10 | 555 | 1.8 | 1064 | 1.9 | 319 | 3.3 | 884 | 3.0 |
| 11 | 685 | 2.9 | 1171 | 2.3 | 68 | 1.8 | 879 | 1.1 |
| x̄ | 755 | 3.7 | 1084[b] | 2.8[a] | 266[c] | 5.0 | 699[d] | 3.0 |
| ± s.d. | ± 206 | ± 2.7 | 303 | ± 1.3 | ± 153 | ± 5.0 | ± 240 | ± 1.7 |

$B_{max}$: binding capacity in fmol mg$^{-1}$ protein; $K_d$: (dissociation constant) in nM; –: not investigated. [a]$P<0.05$, [b]$P<0.01$, [c]$P<0.001$, *vs* the control group (without irradiation). [d]$P<0.01$, *vs* the irradiated control group. Significant ($P<0.01$) increase in $^3$H-PGE$_1$ receptors expressed on human hypernephroma cells was found after pre-treatment with ASA which also led to a significant ($P<0.05$) increase in the binding affinity. Irradiation caused a significant ($P<0.001$) depression of $^3$H-PGE$_1$ receptors without change in the binding affinity. Irradiated ASA-pre-treated cells had a similar binding capacity to the plane control group and a significant ($P<0.01$) higher one as compared with the irradiated control group.

**Table III** $^3$H-PGE$_2$ binding to cultured human hypernephroma cells

| | *Without irradiation* | | | | *Irradiation* | | | |
|---|---|---|---|---|---|---|---|---|
| | *Control (group 1)* | | *ASA (group 2)* | | *Control (group 3)* | | *ASA (group 4)* | |
| *Pat.* | $B_{max}$ | $K_d$ | $B_{max}$ | $K_d$ | $B_{max}$ | $K_d$ | $B_{max}$ | $K_d$ |
| 1 | 563 | 3.0 | 650 | 3.5 | – | – | – | – |
| 3 | 143 | 5.0 | 162 | 1.0 | 63 | 4.6 | 181 | 2.0 |
| 4 | 478 | 6.0 | 858 | 4.0 | – | – | – | – |
| 5 | 650 | 2.0 | 780 | 2.0 | – | – | – | – |
| 6 | 930 | 7.7 | 1104 | 4.6 | 209 | 9.0 | 336 | 6.0 |
| 8 | 438 | 1.0 | 986 | 1.2 | 218 | 2.2 | 338 | 3.0 |
| 9 | 534 | 1.1 | 666 | 1.0 | 112 | 1.2 | 466 | 1.2 |
| 10 | 313 | 5.8 | 324 | 0.3 | 96 | 2.0 | 232 | 3.6 |
| 11 | 398 | 6.3 | 441 | 2.3 | 189 | 9.1 | 170 | 5.0 |
| x̄ | 494 | 4.2 | 663[a] | 2.2[a] | 148[b] | 4.7 | 287[c] | 3.5[c] |
| ± s.d. | ± 221 | ± 2.5 | 309 | ± 1.5 | ± 66 | ± 3.6 | ± 114 | ± 1.8 |

$B_{max}$: binding capacity in fmol mg$^{-1}$ protein; $K_d$: (dissociation constant) in nM; –: not investigated. [a]$P<0.05$, [b]$P<0.01$, *vs* the control group (without irradiation). [c]$P<0.05$, *vs* the irradiated control group. Significant ($P<0.05$) increase in $^3$H-PGE$_2$ receptors expressed on human hypernephroma cells was found after pre-treatment with ASA which also led to a significant ($P<0.05$) increase in the binding affinity. Irradiation caused a significant ($P<0.01$) depression of $^3$H-PGE$_2$ receptors without change in the binding affinity. Irradiated ASA-pretreated cells had a significantly ($P<0.05$) higher number of $^3$H-PGE$_2$ receptors as irradiated ASA-untreated cells, however values were still significantly ($P<0.05$) lower than in the control group.

**Figure 4** a–c Effects of aspirin and irradiation on cAMP-production of hypernephroma cells stimulated by different concentrations of PGs (iloprost **a**, $PGE_1$ **b**, $PGE_2$ **c**). Values indicate the means of four independent experiments. –◇–: control group; –●–: ASA-treated group; –▲–: control group irradiated; –□–: ASA-treated group irradiated. *$P<0.05$, **$P<0.01$ vs the basal value in each group.

**Table IV** Effects of ASA and irradiation on $^{14}C$-AA conversion (in %) by human hypernephroma cells

| Pat. | $TXB_2$ | $PGE_2$ | $PGD_2$ | $PGF_{2\alpha}$ | 12-HETE | AA (%) |
|---|---|---|---|---|---|---|
| *Control group (group 1)* | | | | | | |
| 1 | 8.5 | 12.3 | 2.2 | 2.3 | 4.2 | 65.2 |
| 3 | 6.2 | 11.0 | 2.1 | 2.4 | 3.8 | 58.9 |
| 4 | 11.8 | 20.1 | 3.1 | 3.9 | 4.5 | 49.3 |
| 6 | 7.3 | 7.9 | 3.9 | 4.2 | 3.1 | 68.9 |
| 8 | 5.9 | 9.8 | 4.7 | 2.9 | 4.8 | 62.9 |
| 9 | 8.9 | 15.3 | 5.1 | 3.8 | 3.1 | 59.8 |
| 11 | 7.7 | 7.5 | 3.9 | 2.9 | 2.1 | 67.8 |
| x̄ ± s.d. | 8.0 ± 2.0 | 12.0 ± 4.5 | 3.6 ± 3.6 | 3.2 ± 0.8 | 3.7 ± 1.0 | 61.8 ± 6.7 |
| *ASA-treated group (group 2)* | | | | | | |
| 1 | 1.2 | 1.8 | 1.3 | 1.7 | 6.9 | 66.5 |
| 3 | 0.8 | 1.1 | 0.9 | 0.7 | 4.2 | 78.2 |
| 4 | 1.7 | 2.2 | 0.2 | 0.8 | 7.3 | 72.9 |
| 6 | 1.3 | 0.7 | 0.5 | 0.2 | 5.5 | 78.5 |
| 8 | 1.3 | 1.5 | 0.4 | 0.3 | 5.3 | 82.8 |
| 9 | 2.3 | 1.8 | 0.6 | 0.5 | 4.9 | 81.0 |
| 11 | 1.5 | 1.3 | 0.8 | 0.3 | 8.8 | 78.5 |
| x̄ ± s.d. | $1.4^{c,e}$ ± 0.5 | $1.5^{c,e}$ ± 0.5 | $0.7^{b,e}$ ± 0.4 | $0.6^{c,e}$ ± 0.5 | $6.1^{a,d}$ ± 1.6 | $76.9^{b}$ ± 5.5 |
| *Control group irradiated (group 3)* | | | | | | |
| 1 | 6.6 | 8.3 | 2.9 | 2.1 | 4.6 | 70.4 |
| 4 | 8.2 | 9.3 | 3.1 | 3.9 | 5.2 | 63.2 |
| 6 | 6.9 | 7.8 | 3.3 | 2.5 | 4.3 | 68.9 |
| 9 | 9.1 | 10.5 | 2.9 | 2.6 | 3.6 | 65.2 |
| 11 | 6.4 | 9.3 | 2.9 | 2.4 | 2.8 | 67.5 |
| x̄ ± s.d. | 7.4 ± 1.2 | 9.0 ± 1.0 | 3.0 ± 0.2 | 2.7 ± 0.7 | 4.1 ± 0.9 | 67.0 ± 2.9 |
| *ASA-treated group irradiated (group 4)* | | | | | | |
| 3 | 1.3 | 0.8 | 0.3 | 0.5 | 4.9 | 82.9 |
| 4 | 1.2 | 1.6 | 0.9 | 0.9 | 5.1 | 74.3 |
| 6 | 1.1 | 1.9 | 0.8 | 0.2 | 4.8 | 81.2 |
| 9 | 1.5 | 1.1 | 0.5 | 0.8 | 4.8 | 73.7 |
| 11 | 0.6 | 1.8 | 0.4 | 0.4 | 5.1 | 79.4 |
| x̄ ± s.d. | $1.1^{c,f}$ ± 0.3 | $1.4^{c,f}$ ± 0.5 | $0.6^{f}$ ± 0.3 | $0.6^{c,f}$ ± 0.3 | $4.9^{a}$ ± 0.2 | $78.3^{c,e}$ ± 4.1 |

$^{a}P<0.05$, $^{b}P<0.01$, $^{c}P<0.001$, vs the control group (group 1). $^{d}P<0.05$, $^{e}P<0.01$, $^{f}P<0.001$, vs the irradiated control group.

The presence of PGs as well as PG receptors in cultured human hypernephroma cells suggests a role for PGs in tumour growth and possibly its metastases formation. It has already been reported that certain syndromes of patients with hypernephromas such as the osteolytic process and consecutive hypercalcaemia might result from PGs produced by hypernephroma cells (Robertson *et al.*, 1975; Cummings & Robertson, 1977). Furthermore, increased PG production by tumours has been associated with aggression, whereas inhibitors of PG synthesis including ASA and indomethacin were reported to retard tumour growth and metastases formation (Lynch *et al.*, 1978; Bennett, 1982).

cAMP is a second messenger mediating the actions of PGs and also connected with normal and malignant cell proliferation. It is likely that changes in intracellular cAMP-production due to PG stimulation may greatly influence tumour cell growth and even metastases formation (Heidrick & Ryan, 1971; Sheppard, 1972). However, both reduced cAMP levels (Malachi *et al.*, 1981; Goldberg *et al.*, 1975; Stevens *et al.*, 1978) and elevated cAMP contents (Minton *et al.*, 1976; Küng *et al.*, 1977) have been reported in tumour tissues. This may correlate to the different PG levels found for various tumour tissues. For cultured human hypernephroma cells we found no significant difference in basal cAMP levels in both non-ASA-treated and ASA-treated cells. However, the PG-stimulated cAMP-production was significantly higher in the ASA-treated group as compared with the control group. This suggests that down-regulation of PG receptors was associated with decreased adenylate cyclase activity stimulated by PGs.

We here show that ASA modifies the radioresponse of human hypernephroma cells by preventing the irradiation-induced decrease of PG receptors, which might cause a corresponding decrease in PG-induced membrane adenylate cyclase activity.

The authors are grateful for the expert technical assistance of Ingrid Teufel and Regina Haslinger.

This study was supported by a grant of the 'Kommission für Onkologie', University of Vienna, Vienna, Austria.

### References

BENNETT, A. (1982). Effect of prostaglandin synthesis inhibitors on tumor growth *in vivo*. In *Prostaglandins and Cancer: First International Conference*, Powles, T.J., Bockman, R.S., Honn, K.V. & Ramwell, P. (eds) Vol. 2, pp. 759–766. Alan R. Liss Inc.: New York.

BENNETT, A., CARROLL, M.A., STAMFORD, I.F., WHIMSTER, W.F. & WILLIAMS, F. (1982). Prostaglandins and human lung carcinomas. *Br. J. Cancer*, **46**, 888–893.

CHUN, M. & HOFFMAN, M.K. (1987). Combination immunotherapy of cancer in a mouse model: synergism between tumor necrosis factor and other defence systems. *Cancer Res.*, **47**, 115–118.

CUMMINGS, K.B. & ROBERTSON, R.P. (1977). Prostaglandin: increased production by renal cell carcinoma. *J. Urol.*, **118**, 720–723.

DIERECK, A.M., PRAET, M., ROELS, H., VERBEECK, P., ROBYNS, C. & OOSTERLINCK, W. (1991). Vimetin expression of renal carcinoma in relation to DNA content and histological grading: a combined light microscopic, immunocytochemical and cytophotometrical analysis. *Histopathology*, **18**, 315–322.

FURUTA, Y., HUNTER, N., BARLEY, T. Jr, HALL, E. & MILAS, L. (1988*a*). Increase in radioresponse of murine tumors by treatment with indomethacin. *Cancer Res.*, **48**, 3008–3013.

FURUTA, Y., HALL, E.R., SANDUJA, S., BARKLEY, T. Jr & MILAS, L. (1988*b*). Prostaglandin production by murine tumors as a predictor for therapeutic response to indomethacin. *Cancer Res.*, **48**, 3002–3007.

GOLDBERG, M.L., BURKE, G.C. & MORRIS, H.P. (1975). Cyclic AMP and cyclic GMP content and binding in malignancy. *Biochem. Biophys. Rev. Comm.*, **62**, 320–327.

HALPERIN, E.C. & HARISINDIS, L. (1983). The role of radiation therapy in the management of metastatic renal cell carcinoma. *Cancer*, **51**, 614–617.

HANSON, W.R. & AINSWORTH, E.J. (1985). 16,16-Dimethyl prostaglandin $E_2$ induces radioprotection in murine intestinal and hematopoietic stem cells. *Radiat. Res.*, **103**, 196–203.

HEIDRICK, M.L. & RYAN, W.L. (1971). Adenosine 3′5′-cyclic monophosphate and contact inhibition. *Cancer Res.*, **31**, 1313–1315.

HONN, K.V., BOCKMAN, R.S. & MARNETT, L.J. (1981). Prostaglandins and cancer: a review of tumor initiation through tumor metastasis. *Prostaglandins*, **21**, 833–864.

KÜNG, W., BECHTEL, E., SALOKANGAS, A., PREISZ, J., HUBER, P., TORHORST, T., JUNGMAN, A., KALMADGE, K. & EPPENBERGER, U. (1977). Altered levels of cyclic nucleotides, cyclic AMP phosphodiesterase and adenyl cyclase activities in normal, dysplastic and neoplastic human mammary tissue. *FEBS Lett.*, **82**, 102–106.

LANG, E.K. & DEKERNION, J.B. (1981). Transcatheter embolization of advanced renal cell carcinoma with radioactive seeds. *J. Urol.*, **126**, 581–586.

LYNCH, N.R., CASTES, M., ASTOIN, M. & SALOMON, J.C. (1978). Mechanism of inhibition of tumor by aspirin and indomethacin. *Br. J. Cancer*, **38**, 503–512.

MALACHI, T., CHAIMOFF, Ch., FELLER, N. & HALBRECHT, I. (1981). Prostaglandin $E_2$ and cyclic AMP in tumor and plasma of breast cancer patients. *J. Cancer Res. Clin. Oncol.*, **102**, 71–79.

MILAS, L., FURUTA, Y., HUNTER, N., NISHIGUCHI, I. & RUNKEL, S. (1990). Dependence of indomethacin-induced potentiation of murine tumor radioresponse on tumor host immunocompetence. *Cancer Res.*, **50**, 4473–4477.

MINTON, J.P., MATTEWS, R.H. & WEISENBAUGH, T.W. (1976). Elevated adenosine 3′5′-cyclic monophosphate levels in human and animal tumors *in vivo*. *J. Natl Cancer Inst.*, **57**, 39–41.

PORTEDER, H., MATEJKA, M., ULRICH, W. & SINZINGER, H. (1984). The cyclooxygenase and lipoxygenase pathways in human oral cancer tissue. *J. Max. Fac. Surg.*, **12**, 145–147.

POWLES, T.J., ALEXANDER, P. & MILLER, J.L. (1978). Enhancement of anticancer activity of cytotoxic chemotherapy with protection of normal tissues by inhibition of PG synthesis. *Biochem. Pharmacol.*, **27**, 1389–1392.

ROBERTSON, R.P., BAYLINK, D.J., MARINI, J.J. & ADKISON, H.W. (1975). Elevated prostaglandins and suppressed parathyroid hormone associated with hypercalcemia and renal cell carcinoma. *J. Clin. Endocrinol. Metab.*, **41**, 164–167.

ROBERTSON, R.P., WESTCOTT, K.R., STORM, D.R. & RICE, M.G. (1980). Down-regulation *in vivo* of PGE receptors and adenylate cyclase stimulation. *Am. J. Physiol.*, **239**, E75–E80.

SCATCHARD, G. (1949). The attractions of proteins for small molecules and ions. *Ann. N.Y. Acad. Sci.*, **51**, 660–672.

SHEPPARD, J.R. (1972). Difference in the cyclic adenosine 3′5′-monophosphate levels in normal and transformed cells. *Nature New Biol.*, **236**, 14–16.

SKUBALLA, W. & VORBRÜGGEN, H. (1983). Synthesis of iloprost (ZK36374): a chemically stable and biologically potent prostacyclin analog. *Adv. Prostagl. Thrombox. Leukotr. Res.*, **11**, 299–305.

STEVENS, R.H., LOVEN, D.P., OSBORNE, J.W. & PRALL, J.L. (1978). Cyclic nucleotide concentrations and X-ray induced rat small intestinal cancer. *Cancer Lett.*, **4**, 325–332.

VIRGOLINI, I., HERMAN, M. & SINZINGER, H. (1988). Decrease of prostaglandin $I_2$ binding sites in thyroid cancer. *Br. J. Cancer*, **58**, 584–588.

VIRGOLINI, I., SINZINGER, H., MÜLLER, Ch. & HERMAN, M. (1989). Human hepatocellular cancers show a decreased prostaglandin $E_1$ binding capacity. *Br. J. Cancer*, **59**, 407–409.

VIRGOLINI, I., LI, S.R., SILLABER, Ch., MAJDIC, O., SINZINGER, H., LECHNER, K., BETTELHEIM, P. & VALENT, P. (1992). Characterization of prostaglandin (PG) binding sites expressed on human basophils. Evidence for a $PGE_1$, $PGI_2$ and a $PGD_2$-receptor. *J. Biol. Chem.*, **267**, 12700–12708.

WALDEN, T.L., PATCHEN, J.M. & SNYDER, S.L. (1987). 16,16-Dimethyl prostaglandin $E_2$ increases survival in mice following irradiation. *Radiat. Res.*, **109**, 440–448.

WANDL, E.O., ONO, K., KAIN, R., HERBSTHOFER, T., HIENERT, G. & HÖRBARTH, K. (1989). Linear correlation between surviving fraction and the micronuclear frequency. *Int. J. Radiat. Biol.*, **56**, 771–775.

ZIBOH, V.A., MILLER, A.M., YUNIS, A. & JIMENEZ, J. (1981). Arachidonic acid metabolism by rat chloroleukemia cells in culture. *Cancer Res.*, **41**, 12–17.